# Social capital and health in China: exploring the mediating role of lifestyle

**DOI:** 10.1186/s12889-017-4883-6

**Published:** 2017-11-06

**Authors:** Xindong Xue, Mingmei Cheng

**Affiliations:** 1 School of Public Administration, Zhongnan University of Economics and Law, No.182 South Lake Avenue, East Hi-tech Development Zone, Wuhan, 430073 China; 2 School of Public Finance & Taxation, Zhongnan University of Economics and Law, No.182 South Lake Avenue, East Hi-tech Development Zone, Wuhan, 430073 China

**Keywords:** Individual-level social capital, Social trust, Social relationship, CCP membership, Self-rated health, Psychological well-being, Lifestyle, China

## Abstract

**Background:**

Although social capital as a key determinant of health has been well established in various studies, little is known about how lifestyle factors mediate this relationship. Understanding the cross-relationships between social capital, health, and lifestyle factors is important if health promotion policies are to be effective. The purpose of this study is to explore whether different dimensions of social capital and lifestyle factors are related, and whether lifestyle factors mediate the association between social capital and self-rated health (SRH) and psychological well-being (PWB) in China.

**Methods:**

This study used nationally representative data from the 2014 China Family Panel Studies (*n* = 28,916). The data reported on three dimensions of individual-level social capital: social trust, social relationship and Chinese Communist Party (CCP) membership. Health was assessed using SRH and PWB. Five lifestyle indicators were recorded: healthy diet, physical activity, smoking, sleeping, and non-overweight status. Logistic regression was used to examine the associations between social capital and lifestyle factors, and whether there was a mediating role of lifestyle. Odds ratios relating health status to social capital were reported before and after adjustment for lifestyle factors. Mediation analysis was then used to calculate the total, direct and indirect effects of social capital on SRH and PWB.

**Results:**

The results show that social trust was significantly associated with all five lifestyle factors. Social relationship was significantly associated with four of the five: healthy diet, physical activity, sleeping and non-overweight. CCP membership was only significantly associated with two lifestyle factors: physical activity and non-overweight. Social trust and social relationship were significantly related to both SRH and PWB. CCP membership was only significantly related to SRH. Mediation analysis found modest evidence that lifestyle factors influenced the relationship between all three types of social capital and SRH. In contrast, only social trust and social relationship, but not CCP membership, were mediated by lifestyle factors with respect to PWB.

**Conclusions:**

This study is the first to explore the mediating role of lifestyle factors in the relationship between social capital and health in China. The overall findings suggest that lifestyle factors modestly mediate the association between social capital and health. The degree of mediating effect varies across different dimensions of social capital. Social capital-based health promotion policies would benefit from taking lifestyle factors into account.

## Background

Social capital, defined as “features of social organization, such as trust, norms and networks that can improve the efficiency of society by facilitating coordinated actions” [1], has been identified as an important determinant of health. A robust and positive association between social capital and health has been established in both developed and developing countries [2–7]. In recent years, researchers have directed attention to the causal relationship between social capital and health, with most finding a significant causal effect for the U.S. and many European and Asian countries [8–14].

It has been proposed that social capital affects health through the following channels: promotes more rapid dissemination of information, increases the likelihood of adopting healthy behaviors, and exerts social control over unhealthy behaviors [15]. However, empirical evidence is still lacking on the underlying factors and mechanisms that govern the relationship between social capital and health.

At the conceptual level, social capital is often categorized into cognitive and structural components [16]. Cognitive social capital relates to an individual’s perception of trust, solidarity and reciprocity. Structural social capital refers to the extent and density of social networks, relationships and social participation. In addition to social trust and social relationships, previous studies focusing on China have included Chinese Communist Party (CCP) membership as a structural social capital (social participation) variable [17–19]. The CCP is the ruling party and largest political organization in China, possessing great social and political power. Membership is an important way to access resources relevant to health. In this study, we use social trust, social relationship and CCP membership as individual-level measures of social capital.

It is well-known that lifestyle factors are major determinants of morbidity, mortality and health [20]. The famous “Alameda Seven” study conducted in Alameda County, California in 1965 found that seven lifestyle factors (diet, smoking, exercise, alcohol, sleep, weight and stress) influenced physical health status [21]. Of the seven lifestyle factors, dietary behaviors have been strongly linked with cardiovascular diseases, diabetes and cancers [22]. Physical inactivity has been associated with poor self-rated health [23]. A longitudinal study from the U.K. found that non-smokers had a 10-year longer life expectancy than smokers [24]. Good sleep has been reported to be an important indicator of health and mental well-being [25, 26]. Obesity has been linked to an increased risk of myocardial infarction, stroke, type 2 diabetes, cancer, hypertension, osteoarthritis, asthma, and depression, among other conditions [27, 28]. In summary, the current literature has found that healthy diet, physical activity, non-smoking, good sleeping and non-overweight are positively related to better health.

Relatedly, numerous studies report that social capital is linked to a variety of lifestyle factors. Individuals with poor social relationships are more likely to have a poor diet, smoke, and engage in low levels of physical activity [29–31]. A large population study from England identified positive associations between individual-level social capital (social participation, social trust, and social support) and the choosing of a vegetarian and fruit diet [32]. Likewise, a study in Sweden found a statistically significant association between social participation and fruit and vegetable consumption [33]. Studies among adults in Sweden, the U.S. and Australia found positive associations between social participation and physical activity [34–36]. Social trust has been negatively associated with smoking among Asian Americans [37], Japanese [38, 39], and Germans [40]. In Sweden and England, low social participation and generalized trust have been shown to be associated with daily smoking [32, 41]. Evidence on the relationship between social capital and sleeping is mixed. A study of Japanese and British civil servants reported that social participation was associated with better sleep [42]. However, another study found that the level of social capital, while related to daytime vigilance, was not related to sleeping quality [43].

Generally, those with higher social capital are less likely to be overweight. Findings from Holtgrave and Crosby [44] suggest that greater levels of social capital (social trust, social participation) may deter obesity and diabetes. A study of U.S. adults found that greater community social capital reduced obesity risk [45]. Similarly, a study based on the Austrian Health Interview Survey showed that low social capital (social relationship) was associated with a high risk of being obese [46].

A few studies have investigated the mediating role of lifestyle factors, with mixed results. Poortinga [32] found no mediation effect of lifestyle factors for English subjects. In the Netherlands, physical activity, but not nutrition and sleeping, has been identified as a mediating factor in the relationship between social capital and individual health [47]. A study based on a Finnish health survey observed that part of the association between social participation and networks and health was explained by physical activity [48].

In contrast to the voluminous literature on social capital, lifestyle and health in western countries, relatively little research has focused on China. The objective of this study is to utilize Chinese data to examine, firstly, whether social trust, social relationship and CCP membership are related to lifestyle factors. And, secondly, to examine whether lifestyle factors mediate the relationship between these social capital variables and two measures of health: self-rated health (SRH) and psychological well-being (PWB). To our knowledge, this is the first study to employ a nationally representative, Chinese dataset to examine the mediating role of lifestyle on the relationship between social capital and health. Our hypotheses are: (1) social capital and lifestyle factors are positively related, and 2) lifestyle factors mediate the effects of social capital on self-reported health and psychological well-being in China.

## Methods

### Data set

**Fig. 1:**
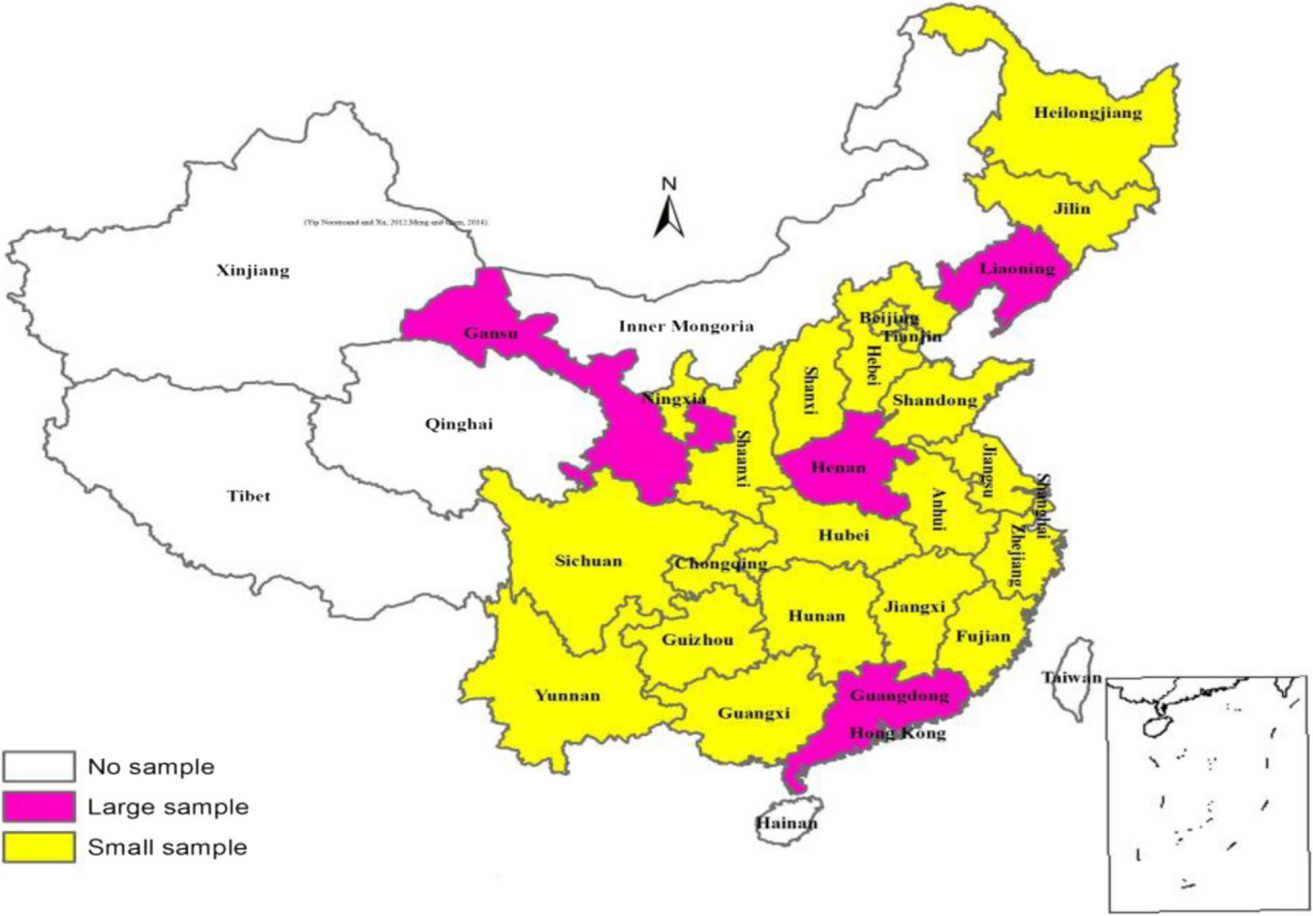
CFPS samples at the Provincial Level. Source: CFPS research team (2016)

This study uses cross-sectional data from the 2014 China Family Panel Studies (CFPS; http://www.isss.edu.cn/cfps/EN/). CFPS is a nationally representative, annual longitudinal survey administered by the Institute of Social Science Survey (ISSS) of Peking University. It includes 37,147 Chinese respondents residing in 621 villages/communities from 25 of China’s 30 provinces [49]. All the sub-sampling frames of CFPS were obtained through a stratified three-stage (districts/counties-villages/communities-households) probability random sampling procedure. The Primary Sampling Unit (PSU) is administrative districts (counties). The second-stage Sampling Unit (SSU) is administrative villages (communities). And the third-stage (Ultimate) Sampling Unit (TSU) is households. Within each household, members aged 16 and above are selected as the respondents. Figure 1 shows CFPS samples at the provincial level.

**Fig. 2:**
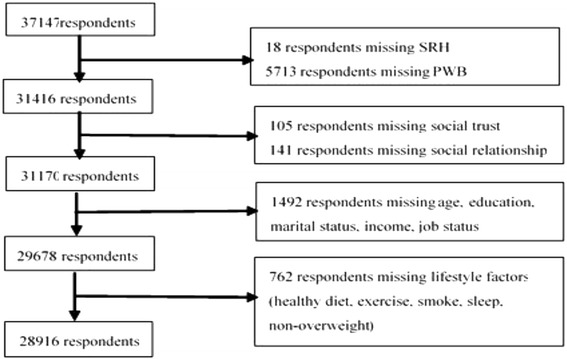
Flow chart on how the final analytical sample was derived (*n* = 28,916)

The data were collected by means of computer-assisted personal interviews (CPI). During all stages of data collection, the research team adopted telephone check, field check, audio record check, interview reviews and statistical analyses to ensure data quality. The survey questionnaire contains detailed individual-, family-, and community-level information on social capital, health, socioeconomic characteristics. This makes the CFPS the ideal dataset for our study. After accounting for missing values in the dataset, our final analytical sample consisted of 28,916 respondents. The overall response rate was 77.84%. Figure 2 provides a flow chart illustrating how the final analytical sample was derived.

### Measurements

#### Health

We use SRH and PWB as health indicators. Previous studies have consistently shown that SRH is a valid and reliable indicator of morbidity and mortality [50]. PWB is associated with physical health outcomes and is an important measure of mental health [51, 52]. In the CFPS, respondents were asked to rate their own health on a five-point scale. The answers were re-coded dichotomously into good (=1, excellent, very good, good) and bad (=0, fair and poor). Psychological well-being was assessed using the 6-item Center for Epidemiologic Studies Depression (CES-D) questionnaire developed by Radloff [53]. Respondents were asked the following six questions about their psychological state over the past month: (1) How frequent do you find it difficult to be excited? (2) How frequent do you feel mental strain? (3) How frequent do you feel restless and cannot keep calm? (4) How frequent do you feel hopeless about the future? (5) How frequent do you find it difficult to do anything? (6) How frequent do you find your life is meaningless? For each of the above questions, the possible answers were: always, almost every day, half the time, sometimes and never, which were assigned 4, 3, 2, 1 and 0, respectively. Scores from each question were summed to obtain an aggregate score ranging between a minimum of 0 (no depression symptoms) and a maximum of 24 (very severe depression symptoms). Higher scores indicate worse psychological health. This variable was further coded dichotomously using a threshold of 6 (good = 1, if score is equal or less than 6, and bad = 0, if score > 6), which was determined by mapping our scale to a threshold of 16 using a 60-point scale, as suggested by Radloff [53].

#### Social capital

As mentioned above, we measured social capital on three different dimensions: social trust, social relationship and CCP membership. Social trust was assessed by asking respondents: “Generally speaking, do you agree that most people are trustworthy?” We coded trust as 1 if the answer was “yes, most are trustworthy”, and 0 if the answer was “we should be as careful as possible”. Social relationship was measured by asking respondents: “How do rate your relationship with your neighbors over the past 12 months?” There are five possible answers: “very harmonious”, “harmonious”, “ordinary”, “sometimes tense” and “very tense”. We then condensed social relationship into a binary variable: 1 (very harmonious and harmonious) and 0 (ordinary, sometimes tense and very tense). CCP Membership was constructed from the question: “are you a member of the following parties or organizations?” If the answer was the CCP, we coded it as 1, otherwise 0.

#### Lifestyle factors

Five lifestyle factors were considered in this study: healthy diet, physical activity, smoking, sleeping, and non-overweight status. All these variables were dichotomized.

Healthy diet was defined according to whether a respondent consumed fresh vegetables and fruits over the past week (1 = yes, 0 = no).

Physical activity was determined by asking whether a respondent did physical activity over the past week (1 = yes, 0 = no).

Smoking status was measured by asking whether a respondent was currently smoking (1 = yes, 0 = no).

Sleeping was based on the question of how many hours a respondent slept during the working days. It was dichotomized as adequate sleep (=1, if no less than 8 h in 24 h) and inadequate sleep (=0, otherwise).

Non-overweight was based on the Body Mass Index (BMI) score which was calculated by weight in kilograms divided by height in meters squared. It was dichotomously coded based on whether a respondent had a score less than 26 (1 = non-overweight, 0 = overweight).

#### Socio-demographic variables

We also controlled a variety of social-demographic variables: age, gender, area of living, education, marital status, family size, income and job status. Age was measured in years. Gender was dichotomously coded as male (=1) and female (=0). Area of living was categorized into urban (=1) and rural (=0). Education was based on number of years of education completed. Marital status was coded into three categories: never married; married or cohabitating; widowed, separated or divorced (WSD). Family size was measured by the number of persons currently living in the household. Income was based on the log transformation of annual, household income per capita. Job status was categorized into four types: other jobs, private business/self-employed, agriculture worker and waged job.

### Statistical analysis

Baron and Kenny [54] recommend that three conditions must be met in mediation analysis. First, the independent variable must affect the mediator. Second, the independent variable must be shown to affect the outcome variables. Third, the mediator must affect the outcome variable. If all these conditions hold in the predicted direction, then adjusting for the mediator will partially or completely attenuate the association between independent and outcome variables.

**Fig. 3:**
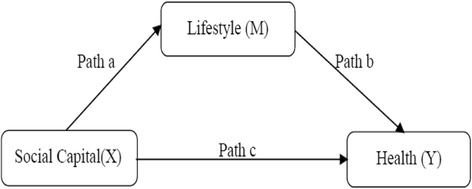
Pathways of direct and indirect effects of social capital (X) through lifestyle (M) on health (Y)

Figure 3 illustrates the direct and indirect effects of social capital variables (X) on a dependent variable (Y) through a mediator variable (M), where path “c” is the direct effect coefficient, and paths “a” and “b” are the indirect effect coefficients. To test the existence and mechanism of association between social capital and health, we performed logistic regression analyses. The first logistic regression analyses were used to determine whether social capital has an effect on lifestyle factors, with separate regressions for each of the lifestyle factors (path a). If all lifestyle factors were significantly related to social capital, they would be used in further analyses. Next, analyses were conducted to determine whether social capital improves health, adjusted for socio-demographic variables (path c). Lifestyle factors were subsequently added to a model containing social capital and socio-demographic variables in order to quantify their contribution to health (path b). Finally, we calculated the coefficients of the total, direct and indirect effects of social capital on health using the *ldecomp* command in Stata. We tested the significance of the mediation with the Sobel test [55]. Given the binary nature of the dependent variables, both direct and indirect effects were standardized [56].

## Results

### Descriptive statistics

In our analytical sample, almost three-fourths of the respondents report good SRH and PWB (71.01% and 73.71%, respectively). The percentage of the respondents who agreed that most people are trustworthy is 53.96%. 74.18% of the respondents have a harmonious social relationship with their neighbors. Only 7.57% of the respondents are CCP members. In terms of socio-demographic variables, age ranges from 16 to 102 with the mean value of 45.7. Half of the sample are males (49.89%) and 47.62% live in an urban area. The mean education level is 7.63 years. Most of the respondents are married (79.94%). The average family size is 4.28. The average logarithm value of annual, household income per capita is 9.09 (about 8127 RMB Yuan). The percentages of private business, agricultural workers, waged job and other jobs are 10.62, 34.53, 30.62 and 24.23%, respectively. With respect to lifestyle variables, most of the respondents have a healthy diet (97.02%). 37% of the respondents did physical activity over the past week. 28.89% currently smoke. Most of the respondents experience good sleep and are not overweight (60.14 and 76.9%, respectively).

### Social capital and lifestyle factors

Table 1 reports the associations between social capital and the individual lifestyle factors, adjusted for socio-demographic characteristics. Each model employs a different behavior as the dependent variable. In Model 1, both social trust and social relationship are positively and significantly related to healthy diet. CCP membership is insignificant. In Model 2, all three social capital variables are positively and significantly related to physical activity. In Model 3, greater social trust is significantly related to a reduced likelihood of smoking. However, social relationship and CCP membership are not significant factors. The results from Model 4 indicate a significant, positive relationship between social trust and social relationship on the one hand, and sleeping on the other. CCP membership is insignificantly related to good sleeping. Finally, while all three social capital measures are significantly linked to being non-overweight in Model 5, only social trust is positively related, with social relationship and CCP membership associated with a higher likelihood of being overweight.

**Table 1:** Odds ratios with 95% confidence intervals of the associations between social capital and lifestyle factors, adjusted for socio-demographic variables

	Model 1: Healthy diet	Model 2: Physical activity	Model 3: Smoking	Model 4: Sleeping	Model 5: Non-overweight
Social capital					
Social trust					
Low	1.00	1.00	1.00	1.00	1.00
High	1.185 (0.994–1.415)*	1.113 (1.044–1.186)***	0.775 (0.724–0.830)***	1.142 (1.080–1.208)***	1.093 (1.029–1.162)***
Social relationship					
Not harmonious	1.00	1.00	1.00	1.00	1.00
Harmonious	1.192(0.998–1.423)*	1.164 (1.079–1.257)***	1.001 (0.921–1.088)	1.066 (0.998–1.140)*	0.880 (0.818–0.946)***
CCP membership					
No	1.00	1.00	1.00	1.00	1.00
Yes	1.051(0.718–1.539)	1.709 (1.535–1.903)***	0.959 (0.853–1.077)	0.948 (0.863–1.043)	0.815 (0.738–0.901)***
Socio-demographic variables					
Gender					
Female	1.00	1.00	1.00	1.00	1.00
Male	0.653 (0.569–0.749)***	1.008 (0.956–1.063)	44.13 (37.62–51.77)***	1.036 (0.985–1.091)	0.835 (0.787–0.887)***
Age in years	1.016 (1.009–1.024)***	1.019 (1.017–1.022)***	1.001 (0.998–1.004)	0.981 (0.979–0.984)***	1.000 (0.998–1.003)
Area of living					
Rural	1.00	1.00	1.00	1.00	1.00
Urban	1.167 (0.881–1.546)	1.474 (1.319–1.646)***	0.903 (0.814–1.003)*	0.822 (0.756–0.895)***	0.813 (0.734–0.901)***
Education in years	1.072 (1.038–1.106)***	1.085 (1.075–1.095)***	0.955 (0.945–0.965)***	0.983 (0.975–0.991)***	1.001 (0.993–1.010)
Marital status					
Never married	1.00	1.00	1.00	1.00	1.00
Married/ cohabitation	0.881 (0.686–1.132)	0.459 (0.415–0.508)***	1.790 (1.581–2.025)***	1.043 (0.950–1.145)	0.305 (0.265–0.351)***
WSD	0.641 (0.451–0.912)**	0.407 (0.351–0.473)***	2.336 (1.933–2.822)***	1.011 (0.876–1.168)	0.383 (0.317–0.462)***
Family size	0.991 (0.938–1.047)	0.963 (0.944–0.982)***	0.989 (0.969–1.010)	1.028 (1.010–1.047)***	1.049 (1.029–1.070)***
Log of income per capita	1.261 (1.180–1.347)***	1.059 (1.023–1.097)***	1.023 (0.990–1.056)	0.961 (0.935–0.988)***	0.920 (0.893–0.949)***
Job status					
Other job types	1.00	1.00	1.00	1.00	1.00
Private business	1.559 (1.132–2.147)***	0.588 (0.527–0.656)***	2.270 (1.987–2.593)***	0.798 (0.719–0.886)***	0.741 (0.667–0.824)***
Agriculture worker	0.891 (0.700–1.133)	0.522 (0.468–0.582)***	2.058 (1.824–2.321)***	0.957 (0.879–1.042)	0.965 (0.872–1.067)
Waged job	0.985 (0.765–1.267)	0.607 (0.558–0.660)***	2.393 (2.150–2.662)***	0.796 (0.734–0.863)***	0.948 (0.864–1.039)

***p < 0.001 **p < 0.05 * *p* < 0.1

### The mediating effects of lifestyle factors

Table 2 presents results for the mediating effects of lifestyle on the associations between social capital and SRH. The basic results without adjusting for socio-demographic variables and lifestyle factors are shown in Model 1. As expected, social trust, social relationship and CCP membership significantly predict better SRH. After adjusting for social-demographic variables (Model 2), the coefficients of the three social capital measures decrease slightly but remain strongly associated with SRH. Model 3 adds the five lifestyle factors. The effects of social trust, social relationship and CCP membership on SRH become weaker, suggesting lifestyle factors play a mediating role in the association between social capital and SRH.

**Table 2:** Odds ratios with 95% confidence intervals of the mediating effect of lifestyle on the association between social capital and SRH

	Model 1	Model 2	Model 3
Social capital			
Social trust			
Low	1.00	1.00	1.00
High	1.263 (1.192–1.338)***	1.204 (1.132–1.281)***	1.195 (1.124–1.271)***
Social relationship			
Not harmonious	1.00	1.00	1.00
Harmonious	1.609 (1.506–1.719)***	1.579 (1.471–1.695)***	1.572 (1.464–1.688)***
CCP membership			
No	1.00	1.00	1.00
Yes	1.071 (0.998–1.185)*	1.130 (1.009–1.264)**	1.109 (0.990–1.242)*
Socio-demographic variables			
Gender			
Female		1.00	1.00
Male		1.360 (1.286–1.438)***	1.321 (1.227–1.422)***
Age in years		0.963 (0.961–0.966)***	0.963 (0.960–0.966)***
Area of living			
Rural		1.00	1.00
Urban		0.988 (0.900–1.085)	0.983 (0.895–1.079)
Education in years		1.043 (1.034–1.052)***	1.041 (1.032–1.050)***
Marital status			
Never married		1.00	1.00
Married/ cohabitation		0.622 (0.539–0.717)***	0.656 (0.568–0.757)***
WSD		0.710 (0.593–0.850)***	0.749 (0.625–0.897)***
Family size		1.035 (1.015–1.055)***	1.034 (1.015–1.054)***
Log of income per capita		1.066 (1.034–1.099)***	1.067 (1.035–1.099)***
Job status			
Other job types		1.00	1.00
Agriculture worker		1.337 (1.186–1.506)***	1.382 (1.227–1.557)***
Private business		1.068 (0.970–1.177)	1.094 (0.993–1.205)*
Waged job		1.229 (1.122–1.347)***	1.258 (1.147–1.380)***
Lifestyle factors			
Healthy diet			
No			1.00
Yes			1.034 (0.828–1.292)
Physical activity			
No			1.00
Yes			1.229 (1.149–1.316)***
Smoking			
Yes			1.00
No			1.062 (0.978–1.152)
Sleeping			
Poor			1.00
Good			1.159 (1.088–1.235)***
Non-overweight			
No			1.00
Yes			1.176 (1.098–1.260)***

***p < 0.001 **p < 0.05 * *p* < 0.1

Table 3 presents the mediating effects of lifestyle factors on the relationship between social capital and PWB. As before, we compare odds ratios as we subsequently add socio-demographic variables (Model 2) and lifestyle factors (Model 3) to the basic model (Model 1). The addition of the latter factors causes the effects of social trust and social relationship on PWB to become smaller. The effects of CCP membership on PWB diminishes to statistical insignificance in Models 2 and 3.

**Table 3:** Odds ratios with 95% confidence intervals of the mediating effect of lifestyle on the association between social capital and PWB

	Model 1	Model 2	Model 3
Social capital			
Social trust			
Low	1.00	1.00	1.00
High	1.491 (1.395–1.593)***	1.440 (1.346–1.541)***	1.425 (1.331–1.524)***
Social relationship			
Not harmonious	1.00	1.00	1.00
Harmonious	1.400 (1.291–1.517)***	1.363 (1.258–1.477)***	1.353 (1.249–1.466)***
CCP membership			
No	1.00	1.00	1.00
Yes	1.299(1.168–1.444)***	1.008 (0.903–1.126)	0.999 (0.895–1.116)
Socio-demographic variables			
Gender			
Female		1.00	1.00
Male		1.336 (1.258–1.419)***	1.402 (1.301–1.510)***
Age in years		0.998 (0.995–1.001)	0.999 (0.996–1.002)
Area of living			
Rural		1.00	1.00
Urban		1.027 (0.912–1.156)	1.027 (0.912–1.157)
Education in years		1.033 (1.023–1.044)***	1.032 (1.022–1.043)***
Marital status			
Never married		1.00	1.00
Married/cohabitation		1.168 (1.045–1.306)***	1.175 (1.049–1.316)***
WSD		0.868 (0.742–1.015)*	0.883 (0.754–1.034)
Family size		1.020 (1.000–1.040)*	1.020 (1.000–1.040)*
Log of income per capita		1.143 (1.108–1.178)***	1.139 (1.105–1.175)***
Job status			
Other job types		1.00	1.00
Agriculture worker		1.081 (0.957–1.222)	1.100 (0.973–1.244)
Private business		0.926 (0.842–1.018)	0.942 (0.856–1.036)
Waged job		1.002 (0.915–1.098)	1.029 (0.939–1.128)
Lifestyle factors			
Healthy diet			
No			1.00
Yes			1.505 (1.234–1.834)***
Physical activity			
No			1.00
Yes			1.065 (0.988–1.147)*
Smoking			
Yes			1.00
No			0.918 (0.844–0.998)**
Sleeping			
Poor			1.00
Good			1.277 (1.199–1.361)***
Non-overweight			
No			1.00
Yes			0.921 (0.856–0.990)**

***p < 0.001 **p < 0.05 * *p* < 0.1

### Total, direct and indirect effects of social capital on health

Table 4 summarizes the standardized coefficients of the total, direct and indirect effects of social capital on SRH (Model 1) and PWB (Model 2) via lifestyle factors. Model 1 shows that social trust, social relationship and CCP membership have significant, indirect effects on SRH through lifestyle factors. These indirect effects account for 8.76%, 6.1% and 24.82% of the total effects, respectively. In Model 2, we find that social trust and social relationship have significant, indirect effects on PWB through lifestyle factors, with the indirect effects accounting for 3.81% and 4.44% of the total effects. However, no significant, indirect effect is found for CCP membership on PWB.

**Table 4:** Total, direct and indirect effects of social capital on health

	Model 1: SRH				Model 2: PWB			
	Coef	Boot S.E	Boot 95% CI	*p*	Coef	Boot S.E	Boot 95% CI	*p*
Social trust								
Total effect	0.194	0.028	0.139–0.250	0.000	0.367	0.027	0.314–0.419	0.000
Direct effect	0.177	0.028	0.122–0.233	0.000	0.352	0.027	0.3–0.405	0.000
Indirect effect	0.017	0.003	0.012–0.022	0.000	0.014	0.003	0.009–0.02	0.000
Indirect effect (%)	8.76%				3.81%			
Social relationship								
Total effect	0.459	0.032	0.397–0.521	0.000	0.315	0.03	0.256–0.374	0.000
Direct effect	0.431	0.032	0.368–0.494	0.000	0.301	0.03	0.241–0.361	0.000
Indirect effect	0.028	0.003	0.022–0.034	0.000	0.014	0.003	0.008–0.02	0.000
Indirect effect (%)	6.1%				4.44%			
CCP membership								
Total effect	0.137	0.057	0.025–0.25	0.017	−0.003	0.06	−0.121-0.114	0.955
Direct effect	0.103	0.057	−0.008-0.214	0.07	−0.001	0.06	−0.119-0.117	0.987
Indirect effect	0.034	0.01	0.015–0.053	0.001	−0.002	0.01	−0,022–0,017	0.811
Indirect effect (%)	24.82%				–			

Adjusted for all socio-demographic variables. Coef = regression coefficient, Boot S.E. = bootstrap standard error used for calculating indirect effects, Boot 95% CI = bootstrap 95% confidence intervals, *p*-values for indirect effects based on Sobel test

## Discussion

This study investigates the relationships between social capital and lifestyle factors with the aim of determining whether lifestyle factors mediate the social capital-health nexus. The results paint a positive, but complex picture of the mediating effects of lifestyle factors.

Our analyses indicate that social trust is the only social capital variable significantly associated with all five types of lifestyle factors when controlling for socio-demographic variables. Those with higher levels of trust were more likely to have a healthy diet, engage in physical activity, be non-smokers, sleep well and non-overweight. These relationships between social trust and lifestyle factors confirm earlier findings for western countries [37–41]. The literature hypothesizes that social trust creates an infrastructure that facilitates the dissemination of health information, while also fostering an unstressed and relaxed environment, which is conducive to the adoption of healthy behaviors and good sleeping.

The results regarding social relationship and CCP membership were less consistent. We found that social relationship was significantly associated with healthy diet, physical activity, sleeping well and being non-overweight, but not with smoking. An unexpected finding is that individuals with good relationships were more likely to be overweight. This stands in contrast to previous studies which indicated that social relationship was a deterrent to obesity [46]. A possible explanation is that, in China, people are more collectively-oriented. Social relationship (especially with neighbors) forms an important part of daily life, and are frequently centered around food and drink. During the process of interaction, social relationship can affect health behavior through peer effects (role model). These behaviors can be good or bad depending on the lifestyle of the neighbors. Some studies have suggested that obesity is contagious through social networks with intimate relations [57]. CCP membership was significantly associated with higher levels of physical activity and lower likelihood of being non-overweight, but it was an insignificant factor in healthy diet, smoking and sleeping. Participation in organizations can be conducive to generating beneficial effects via the transmission of knowledge and increased trust between members of society [58]. However, like social relationship, participation in organizations may also encourage unhealthy behaviors and exert psychological pressure.

All three dimensions of social capital were positively and significantly associated with SRH in our Chinese sample. These results are consistent with previous studies on China [8, 9, 17, 18]. However, while social trust and social relationship were positively and significantly associated with PWB, CCP membership was not significantly related to PWB. It may be that CCP membership provides access to material resources that support physical health, but are not effectual for mental health [17, 18].

Our study also provides fairly modest evidence supporting a mediating role for lifestyle factors. Lifestyle factors were found to mediate the relationship between SRH and all three dimensions of social capital. The intensity of the mediating effects varied from 6.10% to 24.82%, depending on the specific type of social capital. Lifestyle factors also mediated the relationship between PWB and two of the social capital variables: social trust and social relationship. However, no mediating effect was found with respect to CCP membership and health. With respect to existing research on non-Chinese populations, our results stand in contrast to findings by Poortinga [32], but are similar to findings by Mohen [47] and Nieman [48]. More study is needed to explore in greater detail the role that lifestyle factors play in mediating the relationships between social capital and health in China.

### Limitations

This study contains several limitations which may affect the validity of our findings. First, the overall response rate of 77.84% could introduce selection bias, distorting the representativeness of our results. Second, our study is based on cross-sectional data. Cross-sectional data are subject to omitted variable bias where individual, unobserved effects may be correlated with observed variables. Estimated effects would then include the effects from these unobserved factors, which could either magnify or diminish the measurement of true effects. Baron and Kenny [54] identify another concern. The feedback effect between mediator and dependent variable can cause simultaneity bias. For example, while physical activity may predict better SRH, reverse causality can occur whereby sicker people are less able to exercise regularly. Another limitation concerns the level at which we measure social capital. Our analysis focuses on the individual level, but social capital can also be conceptualized and measured at the contextual level [59]. It would be valuable to incorporate both levels simultaneously in analyzing the health effects of social capital. Future research could employ a multilevel approach to separate individual from contextual effects. Most of our measurements of social capital, health and lifestyle factors were evaluated by single-question, self-reported items on a questionnaire. As a result, they may suffer from justification bias and misclassifications. Finally, it may be inappropriate to use CCP membership to assess structural social capital because CCP membership is more prevalent among males and people with higher education. As a result, the estimated effect of this dimension of social capital may not be representative of the larger Chinese population.

## Conclusions

This study is the first to explore the mediating role of lifestyle factors on the relationship between social capital and health in China using a nationally representative data. Our overall findings have important implications for public health policy in China. We provide evidence that lifestyle factors influence the mechanisms linking social capital and health. Strengthening and developing social capital (especially social trust and social relationship) among the Chinese population should be a priority in health promotion. Our results suggest that social capital-based health policies would benefit from taking lifestyle factors into account.
